# Inapparent *Streptococcus agalactiae* infection in adult/commercial tilapia

**DOI:** 10.1038/srep26319

**Published:** 2016-05-24

**Authors:** Jiufeng Sun, Wei Fang, Bixia Ke, Dongmei He, Yuheng Liang, Dan Ning, Hailing Tan, Hualin Peng, Yunxin Wang, Yazhou Ma, Changwen Ke, Xiaoling Deng

**Affiliations:** 1 Guangdong Provincial Institute of Public Health, Guangdong Provincial Center for Disease Control and Prevention. Guangzhou, Guangdong 511430, China; 2 Guangdong Provincial Center for Aquatic Animal Disease Control and Prevention. Guangzhou, Guangdong, 510222, China; 3 Guangdong Provincial Center for Disease Control and Prevention. Guangzhou, Guangdong 511430, China.

## Abstract

We report on inapparent infections in adult/commercial tilapia in major tilapia fish farms in Guangdong. A total of 146 suspected isolates were confirmed to be *S. agalactiae* using an API 20 Strep system and specific PCR amplification. All isolates were identified as serotype Ia using multiplex serotyping PCR. An MLST assay showed single alleles of *adhP* (10), *atr* (2), *glcK* (2), *glnA* (1), *pheS* (1), *sdhA* (3) and *tkt* (2), and this profile was designated ‘unique ST 7’. The analysis of virulence genes resulted in 10 clusters, of which *dltr*-*bca*-*sodA*-*spb1*-*cfb*-*bac* (62, 42.47%) was the predominant virulence gene profile. The PFGE analysis of *S. agalactiae* yielded 6 distinct PFGE types (A, B, C, D, F and G), of which Pattern C (103) was the predominant type, accounting for approximately 70.55% (103/146) of the total *S. agalactiae* strains. Therefore, unlike what has been found in juvenile tilapia, in which PFGE pattern D/F is the major prevalent pattern, we found that pattern C was the major prevalent pattern in inapparent infected adult/commercial tilapia in Guangdong, China. In conclusion, we close a gap in the current understanding of *S. agalactiae* epidemiology and propose that researchers should be alert for inapparent *S. agalactiae* infections in adult/commercial tilapia to prevent a potential threat to food safety.

*Streptococcus agalactiae* (Lancefield group B; GBS) was first recognized as an important opportunistic agent of humans in the mid-twentieth century^1^,^2^ since the first report in 1939^3^. Except for *S. agalactiae*, which colonizes the gastrointestinal tracts of healthy adults^1^,^4^ and the mammary glands of various ruminants^5^,^6^, invasive infection by *S. agalactiae* occurs only in particular individuals, such as pregnant women, infants, the elderly and immunocompromised adults. Over the past few decades, *S. agalactiae* has frequently been isolated from a variety of non-human sources and is now well recognized as an important causative agent of zoonosis^5^,^7^,^8^,^9^,^10^. Recently, continuous outbreaks of streptococci infections by *S. agalactiae* have been reported in various fishes. These outbreaks result in a loss of 40 million dollars annually^11^,^12^. Hence, the authorities now realize that these infections pose a large threat to the fish farming industry, particularly the tilapia industry^11^,^13^,^14^,^15^, because of an increasing requirement for the production, consumption and trading of tilapia around the world.

In China, *S. agalactiae* infections have caused sporadic outbreaks in tilapia fish farms in Guangdong, Guangxi, Hainan and Fujian^16^,^17^,^18^,^19^ that have led to high cumulative mortality rates in fish on individual farms^12^,^20^. The genetic diversity of *S. agalactiae* strains derived from humans, cows and tilapia has been analysed using a broad range of genomic techniques, such as RAPD, PFGE and MLST^21^,^22^,^23^,^24^. However, the results have varied, and only PFGE has been shown to provide sufficient discriminatory power to distinguish between different clone lineages. Although previous studies have reported the partial molecular epidemiological characteristics of this infective agent, most isolates have been obtained from acute infections in juvenile tilapia. In particular, it remains unclear whether inapparent infections exist in adult/commercial tilapia. To close this gap in our knowledge of *S. agalactiae* epidemiology, 146 strains were isolated from adult/commercial tilapia that were obtained from major tilapia fish farms in Guangdong from August to December 2014. The molecular epidemiological characteristics of these strains were analysed using serotyping, MLST, virulence gene profiles determination, PFGE genotyping and environmental stress tests.

## Materials and Methods

### Ethics statement

The protocol of tilapia sampling was approved by the Ethics Committee of the Guangdong Provincial Center for Disease Control and Prevention (Guangdong CDC). The animal experiments in this study were carried out in strict accordance with the recommendations in the Guide for the Care and Use of Laboratory Animals of the Ministry of National Institutes of Health (GB 14922.2-2011). All procedures for sampling and data collection methods were performed in accordance with above regulations.

### Sample collection

**Figure 1:**
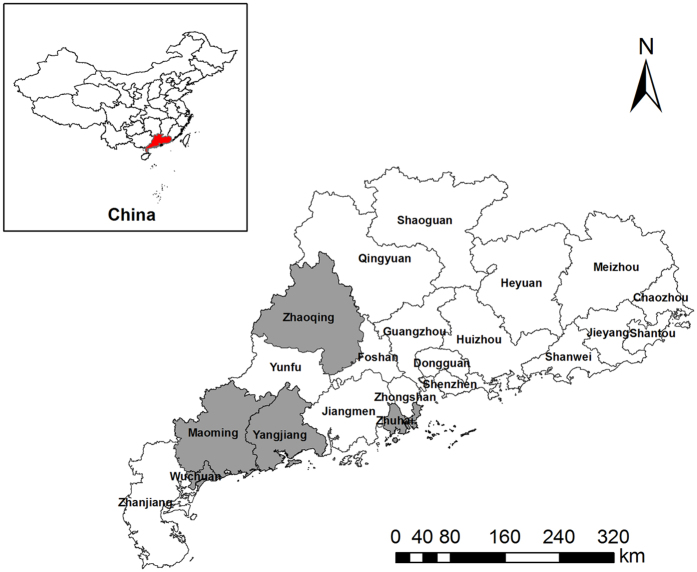
Locations from which tilapia were sampled in Guangdong. The 5 isolation sites are marked in Guangdong Province, China. The location of Guangdong is also shown in the figure. The geographical map was generated using ArcGIS software (version 10.2, Environmental Systems Research Institute, Inc., Redlands, USA) (www.esri.com).

From August to December 2014, adult/commercial tilapia (2–3 kg) with no clinical symptoms (e.g., exophthalmia, corneal opacity, swimming abnormalities and melanosis) were collected from four major tilapia cultivation areas in the Guangdong Province of China. A total of 10 farms in 5 districts in Guangdong Province were investigated and sampled (Fig. 1). There was no exchanging of fish between the sampled fish farms. The young tilapia were also obtained from different sources. For each farm, the geographical origin data of each isolate was recorded for further analysis.

### Isolation of causative strains

Tissue samples were directly and aseptically collected from lesions in the muscle of the tilapia and inoculated onto blood agar plates (Dijing, Guangzhou, China). The plates were incubated at 28 °C for 24 h. A suspected single colony from each initial plate was transferred to a new blood agar plate to obtain pure cultures. Isolates were identified using the API 20 Strep system (Bio Merieux, France) according to the manufacturer’s instructions^25^.

### Identification of suspected isolates by specific PCR amplification

Briefly, all isolates were incubated on blood agar plates (Dijing, China) at 28 °C for 24 h. Genomic DNA was extracted from each isolate using a Gentra Puregene Yeast/Bact Kit (Qiagen, Germany) according to the manufacturer’s instructions. DNA concentrations were measured spectrophotometrically at 260 nm (Shimadzu Corp., Kyoto, Japan). A 2.0 μl solution containing DNA (0.5–1 μg) was used as a PCR template. The duplex-PCR used in this study was referred to in a previous study that targeted the *cfb* gene (CAMP factor gene) of *S. agalactiae* and the 16S rDNA of *S. iniae*^16^. The primer sequences that were used are listed in Table 1. *S. iniae* (ATCC29178), *S. agalactiae* (ATCC27956), and *S. dysgalactiae* (NCTC4335S) were used as reference strains. Each PCR mixture (25 μl) contained 12.5 μl of 2 × GoTaq Mix (Promega, USA), 1.0 μl of each 10 mM primer, 2.0 μl of template DNA and 6.5 μl of dH_2_O. PCR amplification was performed in a GeneAmp PCR system 9700 (ABI, USA). After an initial denaturation at 94 °C for 5 min, the following thermocycling parameters were used for the duplex-specific primers: 35 cycles of 94 °C for 30 s, 58 °C for 30 s and 72 °C for 45 s, and a final extension at 72 °C for 7 min. The PCR amplicons were analysed using electrophoresis in 1.0% agarose gels, and photos were taken using an electrophoresis-photography system (Bio-Rad, USA). The *S. agalactiae* strains were expected to show a 474 bp fragment that corresponded to a part of the *cfb* gene, while a 296 bp region of the 16S rDNA was amplified from *S. iniae*. No genes were expected to be amplified from the reference strains, *S. agalactiae* and *S. dysgalactiae*, because of the specificity of the primers.

### Serotyping by multiplex PCR

The serotype of each isolate was determined using a multiplex PCR assay that targeted capsular genes, as was previously reported by Imperi *et al.*^26^. The primers that were used are listed in Table 2. Five microliters of a DNA solution was used as the template in a final 25 μl PCR mixture that contained the following: 2 mM MgCl_2_ PCR buffer, 200 μM of dNTPs, 250 nM of primers (except for primers 1 and 16, which were used at a concentration of 400 nM), and 0.3 U of HotStart Taq DNA polymerase (TaKaRa, China). The samples were amplified via denaturation for 5 min at 95 °C, followed by 15 cycles at 95 °C for 60 s, 54 °C for 60 s and 72 °C for 2 min, an additional 25 cycles at 95 °C for 60 s, 56 °C for 60 s, and 72 °C for 2 min, and a final cycle at 72 °C for 7 min. The PCR amplicons were analysed using electrophoresis in 1.5% agarose gels. By using GBS reference strains that represented all recognized serotypes, UV transillumination of the amplified products on the agarose gels showed a two or three band pattern, each of which was specific to and characteristic of each serotype^26^. A 688 bp band corresponded to a conserved fragment of the *cpsL* gene (capsular gene L), which was used as an internal positive control. The standard amplicon pattern for serotype Ia to IX were 272 bp and 688 bp; 272 bp, 621 bp, and 688 bp; 272 bp, 465 bp, and 688 bp; 352 bp and 688 bp; 272 bp, 538 bp, and 688 bp; 272 bp, 582 bp, and 688 bp; 352 bp, 470 bp, and 688 bp; 179 bp, 272 bp, and 688 bp; 438 bp and 688 bp; and 229 bp, 272 bp, and 688 bp, respectively.

### MLST

Multilocus sequence typing (MLST) was performed as previously described^27^. Briefly, PCR was used to amplify partial fragments of seven housekeeping genes (*adhP*, *pheS*, *atr*, *glnA*, *sdhA*, *glcK*, and *tkt*). All primer sequences that were amplified for sequencing were obtained from the MLST Database (http://sagalactiae.mlst.net). PCR reactions were prepared by combining 2 μl of isolated DNA with PCR buffer containing a final concentration of 1.5 mM MgCl_2_ (ABI, USA), 0.2 mM of each dNTP (Promega, USA), 0.2 mM of the appropriate forward and reverse primer and 1.25 U of GoTaq DNA polymerase (Promega, USA). The PCR products were purified and sequenced. An allele number was assigned to each fragment after it was aligned with the sequence in the online database (http://sagalactiae.mlst.net). Each isolate was assigned a sequence type (ST) based on the allelic profile of the seven amplicons.

The ST most likely to be the founder of the clonal complex was determined using eBURST software version 3, as recommended previously^28^. In addition, we used a stringent group definition wherein isolates with more than five matching housekeeping alleles were placed in the same complex. All of the reliable STs that belonged to *S. agalactiae* according to the website (http://sagalactiae.mlst.net) were used to construct this network (1376 isolates, 308 STs) (Table S1).

### Determination of virulence genes

The isolates were investigated to identify the following genes that encode surface-localized proteins: surface protein of GBS (*spb1*), C5a peptidase (*scpB*), α- (*bca*) and β-subunits (*bac*) of C protein, regulatory protein (*dltR*), toxins CAMP factor (*cfb*) and superoxide dismutase (*sodA*) in PCR assays using primers and conditions that have been published previously^29^ (Table 3).

### PFGE

PFGE was performed according to a previously published protocol^12^,^16^. Briefly, strains were grown overnight on 5.0% blood agar plates. The cells were harvested and washed two times with solution buffer (Tris-HCl, 0.01 M; EDTA, 0.1 M; pH 8.0). Streptococcus cells were lysed with 1.0 mg/mL lysozyme and 1.0 mg/mL proteinase K (Sigma, USA). The bacterial suspensions were mixed with an equal volume of 1.0% low-melting-point agarose (Cambrex, USA) and pipetted into a 100 μl plug. A solution in CLB (50 mM Tris, 50 mM EDTA, 1% SDS, 0.1 mg/mL proteinase K) was then added. The plugs were incubated in a solution with 12 U of *SmaI* restriction enzyme (Takara, China) and its associated buffers and then then sent for PFGE assay using the following program: a switch time of 4–40 s, 20 h, a 120° angle and a voltage gradient of 6 V/cm in a CHEF Mapper XA (Bio-Rad, USA). A lambda ladder PFGE marker (New England Biolabs, USA) was used as a DNA size marker. The gels were stained with ethidium bromide and photographed under UV light. PFGE patterns were then analysed and compared using BioNumerics version 6.5 software (Applied Maths BVBA, Belgium).The unweighted-pair group method was used with arithmetic averages and Dice’s coefficient in the UPGMA Programme to process the data.

### Resistance to osmotic pressure, acetic acid and temperature

The representative strains within each dominant PFGE pattern were randomly selected and inoculated on Brain Heart Infusion agar (BHI; OXOID, Basingstoke, UK) plates supplemented with NaCL (0, 5, 10, 20, 30, 40 or 50 g/L) or acetic acid (1, 3, 5, 10, 20 or 30%, v/v). The plates were incubated at 28 °C for 12 h. The resistance to cardinal growth temperatures was evaluated in cells grown on BHI plates at 45 °C and 65 °C for 12 h. In addition, the pure cultures were incubated at 80 °C, 90 °C or 95 °C for 1 min and then 28 °C for 12 h. Growth was evaluated as either present or absent.

## Results

### Strain isolation, identification, serotyping and geographical distribution

**Figure 2:**
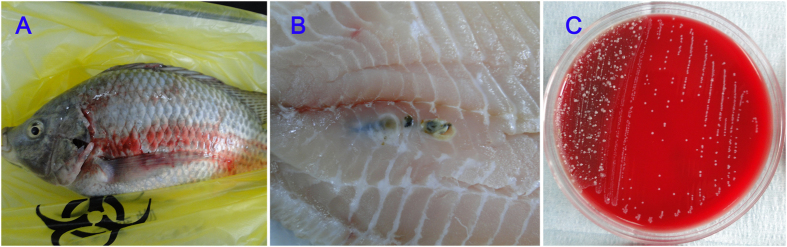
Adult tilapia (**A**), lesions in tissue samples (**B**) and positive cultures grown on blood medium (**C**).

Tissue samples from adult/commercial fish (2–3 kg) were inoculated onto blood agar plates for strain isolation, and suspected colonies were sampled for further purification cultures in the same medium (Fig. 2). A total of 172 suspected isolates were obtained from 229 samples. One hundred and forty-six suspected isolates were confirmed to be *S. agalactiae* according to the biochemical profiles of the strains, which were obtained using an API 20 Strep system (Table S2). All 146 of the biochemical assays confirmed that the *S. agalactiae* strains showed a 474 bp amplicon that corresponded to a part of *cfb* gene of *S. agalactiae* following specific PCR amplification. The specificity of the PCR assay was demonstrated by the fact that no specific band was amplified from either the reference strains or the blank control.

**Figure 3:**
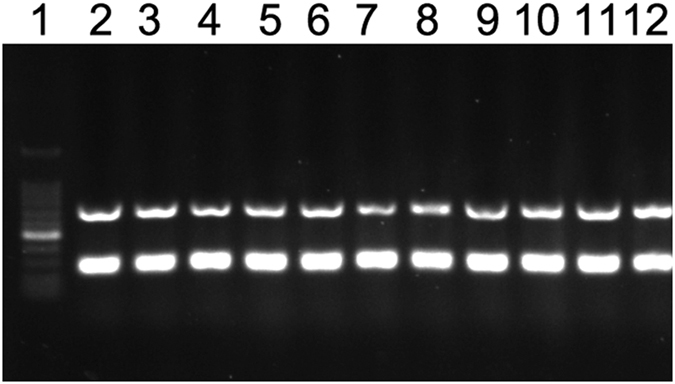
Gel electrophoresis was performed to obtain multiplex PCR amplification products from partial *S. agalactiae* isolates. Direct analysis of amplicon sizes and band patterns allowed the determination of the molecular serotypes of the strains, as follows: lane 1, the molecular marker, a 100 bp DNA ladder; lane 2, positive control (ATCC27956, Ia); lanes 3-12, randomly selected isolates from 146 *S. agalactiae* strains.

Multiplex serotyping PCR was performed on the 146 *S. agalactiae* strains, and the results showed the same amplicons patterns, 272 bp and 688 bp, which corresponded to *S. agalactiae* serotype Ia, according to the standard amplicon patterns described in the Imperi *et al.* study^26^ (Fig. 3). The geographical distribution assay showed that 52, 49, 21 and 24 *S. agalactiae* strains were isolated from samples obtained from Maoming, Zhuhai, Wuchuan and Yangjiang in Guangdong, respectively. No *S. agalactiae* strains were isolated from the samples from Zhaoqing, Guangdong. The rate at which strains were positively isolated from each area varied between 57.65% and 80.00% in this study (Table 4).

### MLST

MLST was applied to all 146 of the *S. agalactiae* strains by amplifying and sequencing PCR fragments for seven housekeeping genes (*adhP*, *atr*, *glcK*, *glnA*, *pheS*, *sdhA* and *tkt*), as previously described^27^. All seven genes were successfully amplified from each isolated strain. After trimming was performed using SeqMan (DNAStar, Madison, WI), the consensus sequences were searched to identify the ST against the GBS database (http://pubmlst.org/sagalactiae). The results showed that all of the strains belonged to seven existing alleles, including *adhP* (10), *atr* (2), *glcK* (2), *glnA* (1), *pheS* (1), *sdhA* (3) and *tkt* (2), and they were therefore designated ‘unique ST 7’. No polymorphic sites were identified within the 146 *S. agalactiae* strains.

**Figure 4:**
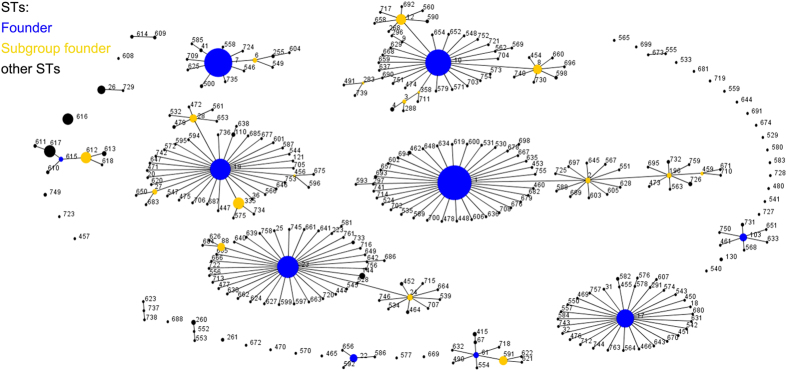
The eBURST illustration that was generated using MLST international database data. No. of isolates = 1376, no. of STs = 308, no. of re-samplings for bootstrapping = 1000, and no. of loci per isolate = 7. Founding and subgroup founding genotypes are shown in blue and yellow, respectively. The size of the dots is representative of the number of isolates belonging to that ST.

The eBURST tool infers patterns of evolutionary descent among clusters of related genotypes from MLST data and identifies mutually exclusive groups of related genotypes within populations. Initial analyses using eBURST revealed the presence of spatial differentiation among the 308 STs that were available in the MLST international database (http://pubmlst.org/sagalactiae) (Fig. 4). Widespread relatedness was demonstrated within the 1376 isolates, and shown by the grouping of the majority of STs into eBURST groups, which were connected by pair-wise identities for five or six of the seven gene fragments, indicating that they share five or six of the seven alleles that define the ST. In other bacterial species, these groups are also referred to as “Clonal Complexes” or “ST Complexes”. In total, 1376 isolates were assigned to 10 Clonal Complexes (CC) (CC 1, 7, 10, 17, 19, 22, 23, 61, 103 and CC 615). ST 7, the unique ST among the isolates obtained from adult/commercial tilapia in Guangdong, belonged to the ST 7 Clonal Complex (CC7) which comprised 14 STs (n = 183) that are distributed around the world (Fig. 3). ST 7 (n = 164) was recognized as the founder of CC 7. The other 13 STs shared five or six of the seven ST 7 alleles, including 10 Single Locus Variants (SLVs; ST 6, 41, 255, 500, 546, 558, 604, 625, 709 and 735) and 3 Double Loci Variants (DLVs; ST 549, 585 and 728). Comparative geographical analysis showed that the CC 7 isolates came from more than ten countries that are distributed throughout Europe and Asia. The original sources were also diverse, including human samples (blood and vaginal swab) and non-human materials (cow milk, ear swab, and fish tissue) (Table S1). In addition, the 157 CC 7 isolates were assigned to four serotypes, including Ia (n = 151), Ib (n = 4), III (n = 1), and IV (n = 1). The isolates in serotype Ia (n = 177) were mainly isolated from non-human samples (tilapia and cow milk), while serotype Ib (n = 4), III (n = 1) and IV (n = 1) were all isolated from human samples that were obtained from either normal carriers or patients with bacteraemia (Table S1).

### Determination of virulence genes

Five genes, including *dltr* (146, 100%), *bca* (146, 100%), *sodA* (145, 99.31%), *spb1* (143, 97.95%), and *bca* (141, 96.58%), were frequently detected in most of the *S. agalactiae* strains, making them the dominant virulence genes in these isolates. The *cfb* (99, 67.81%) and *scpb* genes were detected in 67.81% (n = 99) and 32.99% (n = 48) of 146 isolates, respectively. All 146 isolates were grouped into 10 clusters according to the number of virulence genes they contained (Table 5). The *dltr*-*bca*-*sodA*-*spb1*-*cfb*-*bac* (62, 42.47%), *dltr*-*bca*-*sodA*-*spb1*-*cfb*-*bac*-*scpb* (36, 26.47%), *dltr*-*bca*-*sodA*-*spb1*-*bac* (30, 20.55%) and *dltr*-*bca*-*sodA*-*spb1*-*bac*-*scpb* (9, 6.16%) clusters were the predominant virulence gene patterns. Thirty-six isolates were positive for all 7 virulence genes.

### PFGE

**Figure 5:**
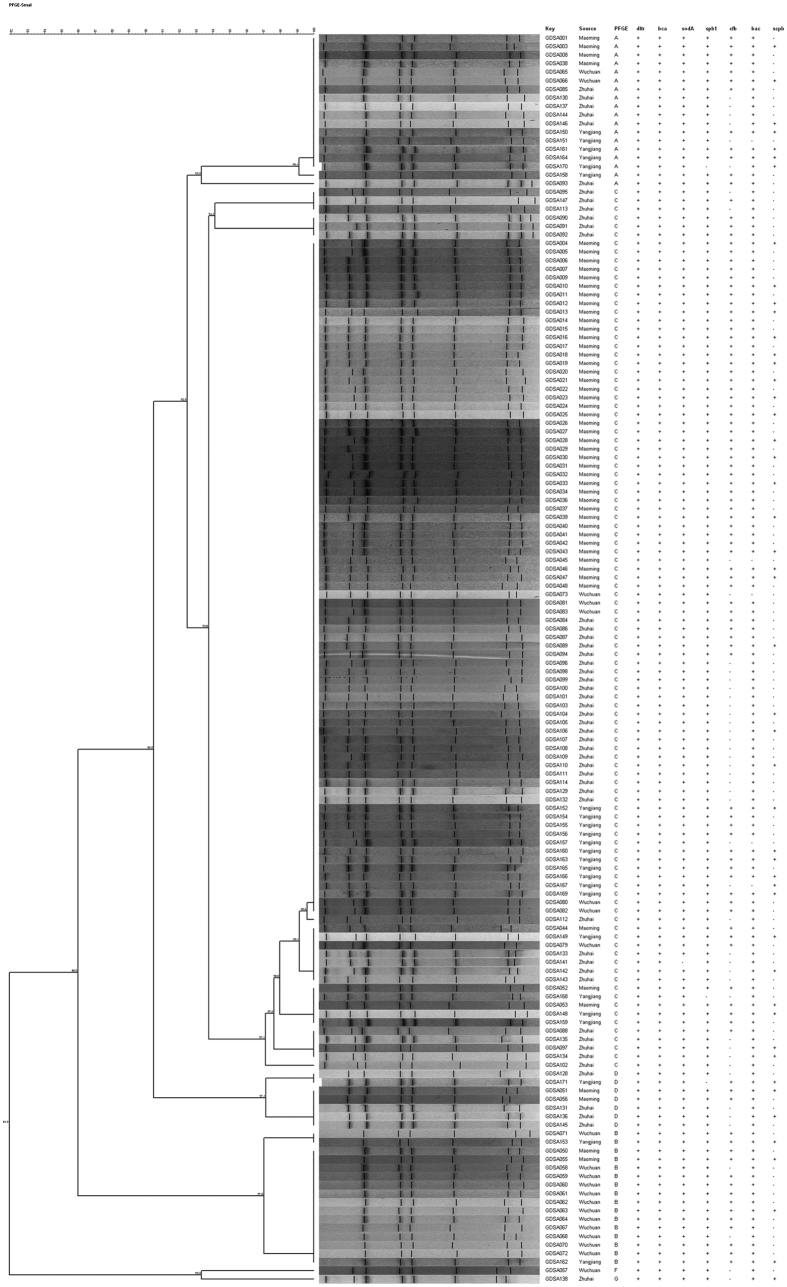
The PFGE dendrogram that was constructed using similarity and clustering analyses for the 146 *S. agalactiae* strains that were isolated from tilapia grown in concentrated aquaculture areas in China. The digitalized genotyping patterns were analysed using the Dice coefficient and UPGMA methods.

PFGE was performed using *S. agalactiae* chromosomal DNA that was digested using *SmaI*. This yielded 6–11 fragments in the 10–85 kb size range (Fig. 5). The 146 *S. agalactiae* strains were distributed among 6 distinct PFGE types (A, B, C, D, F and G), and the similarity among these was between 86% and 100%. Patterns A (n = 18), B (n = 16), C (n = 103) and D (n = 7) were the predominant types, accounting for approximately 98.63% (144/146) of all of the *S. agalactiae* strains. The geographical analysis found that isolates with PFGE patterns A and C were isolated from all four sampling areas. The isolates with pattern B were distributed in Wuchuan, Maoming and Yangjiang, whereas the isolates with pattern D were mainly isolated from Maoming, Zhuhai and Yangjiang. The unique isolates with patterns F and G were isolated from Maoming and Zhuhai, respectively. Surprisingly, we found that a number of clonal strains showed high similarity with PFGE pattern A, B, C and D. In particular, the Pattern C isolates from different fish farms showed 100% similarity with each other. In addition, unlike previous studies showing that the PFGE D/F pattern was the major prevalent pattern in juvenile tilapia, we found that PFGE pattern C was the major prevalent pattern in inapparent infected adult/commercial tilapia in Guangdong, China (Table S2, Fig. 5). However, we did not find a correlation between PFGE and MLST results because of the insufficient discrimination power of MLST compared to that of the PFGE analysis in this study.

The joint analysis of virulence profiles and PFGE patterns showed that the virulence profiles corresponded to the dominant PFGE patterns. The *dltr*-*bca*-*sodA*-*spb1*-*cfb*-*bac* (62, 42.47%), *dltr*-*bca*-*sodA*-*spb1*-*cfb*-*bac*-*scpb* (36, 26.47%) and *dltr*-*bca*-*sodA*-*spb1*-*bac* (30, 20.55%) virulence gene patterns accounted for 87.67% of the isolates with four PFGE patterns (Table 5, Fig. 5).

### Resistance to osmotic pressure, acetic acid and temperature

The representative strains with PFGE pattern C showed strong resistance to osmotic pressure (40 g/L) and temperature (65 °C), followed by patterns D, A and B (Table 6). No differences were observed following treatment at 80 °C, 90 °C or 95 °C for 1 min. However, we found that the isolates with pattern B were more resistant than those with patterns A, C or D when the cells were cultured on BHI with 1% acetic acid, whereas there was no growth in any of the isolates grown on BHI medium with 3–30% acetic acid (Table 6).

## Discussion

*S. agalactiae* is known to be an important opportunistic agent in humans since the last century. Normally, it is present as a commensal organism that is carried by up to 50% of healthy adults, in whom it causes clinical and sub-clinical symptoms. Recently, it has been recognized as a serious causative agent of zoonosis. Cross-infection with *S. agalactiae* between humans, cattle, mice, lizards and tilapia has been frequently reported. In particular, the continuous outbreaks of *S. agalactiae* infection in tilapia fish farms seriously threaten the safety of the tilapia industry and the health of occupational workers and consumers. In North America and Asia, before 2008^30^,^31^,^32^,^33^, a number of cases had already been reported in which humans were infected with *S. iniae* through direct contact with diseased tilapia. Although there has been no report of humans being infected with *S. agalactiae* through contact with diseased tilapia, we need to be alert for inapparent *S. agalactiae* infection in these fish. Previous studies have focused mainly on acute infections in juvenile tilapia. In fact, the threat of inapparent infection in adult/commercial tilapia is more serious than the treat of acute infection in juvenile tilapia because of the higher economic burden these infections place on fish farmers and the potential threat they present to public food safety. However, there is a major gap in our understanding of the epidemiology of *S. agalactiae* inapparent infections in adult/commercial tilapia.

Guangdong is one of the main tilapia-producing provinces in China, along with the Guangxi, Hainan and Fujian provinces, which account for 40% of the total production of tilapia in worldwide. Unfortunately, sporadic outbreaks of tilapia infections caused by *S. agalactiae* have been continuously reported in fish farms in the above provinces in recent years^16^,^17^,^18^,^19^, and these infections have led to high cumulative mortality rates in fish at individual farms^12^,^20^. Seasonal outbreaks of tilapia infections occur mainly in juvenile tilapia from May to October, when water body temperatures are high (25–37 °C)^34^. In this study, 229 adult/commercial tilapia tissue samples were taken at the end of 2014 from 10 individual farms that belonged to five main tilapia farms. After the strains were identified using biochemical profiles and specific PCR amplification, one hundred and forty-six suspected strains were confirmed to be *S. agalactiae*, but no *S. iniae* or any other streptococcus species were identified. These results are not surprising because of the shift in the prevalent strains that was observed in tilapia streptococci from *S. iniae* to *S. agalactiae* in 2008^16^,^35^. It has been proposed that the rare and disastrous cold weather in 2008 caused this dramatic species shift^16^.

There are ten serotypes, which are referred to as Ia, Ib, and II-IX subdivided by GBS capsular polysaccharides, within *S. agalactiae*. The epidemiological distribution of these serotypes can vary according to several factors, including geographical distribution, host and sampling source^36^. No direct correlation has been demonstrated between particular serotypes and sampling sources in previous studies, but the distribution of serotypes of invasive *S. agalactiae* isolates that were derived from vaginal swabs were significantly different from those of colonizing isolates obtained from newborns^37^. The unique serotype Ia that was detected in this study has spread worldwide and has been isolated from both human and non-human samples. In addition, it was the major serotype that was isolated from tilapia infections in a variety of geographical regions of European and Asian countries. The manifestation of the disease that is caused by serotype Ia also varies from sub-clinical in carriers to bacteraemia in humans and invasive infections in non-human animals, such as cow and tilapia (Table S1).

The subsequent MLST analysis showed that all of the isolates belonged to ST 7, which was assigned to CC 7 with a medium genetic exchange rate. Although CC 7 is different from the clinical-specific CC 17 (which includes ST 17, 31 and 148) and the bovine-specific CC 61 (ST 61, 76 and 91), the CC 7 (ST 7) founder has been frequently isolated from tilapia, bovine and humans in Japan and China^27^. The archived information from the MLST database showed that ST 7 is the most prevalent ST in CC 7. Surprisingly, we did not observe the other 13 STs within CC 7. These observations were unexpected given the increasing trade in tilapia between Guangdong and other countries, suggesting that there are geographic-specific factors that affect the prevalence of particular STs and possibly indicating that different dynamics govern the spread of individual STs. A search of the allele profiles in the MLST international database revealed that the other 13 STs were SLVs or DLVs of ST 7. These data indicated that these strains have probably undergone divergent evolution to expand from a founder lineage as a result of a recent genetic event (e.g., mutation or recombination). Therefore, it is believed that the STs in CC 7 have different genetic backgrounds that may be reflected as distinct gene gain/loss events in the genome and that result in different genetic profiles, e.g. virulence genes. Surprisingly, the results of the virulence profile assays of the 146 isolates showed that all of them were assigned to 10 virulence gene profiles and that 4 of them accounted for 87.67% of the 146 isolates. These data probably indicate that ST 7 itself is still expanding as a result of potential mutation or recombination events.

The further PFGE data revealed that the dominant genotypes of streptococci varied between different areas in Guangdong. Pattern C (70.55%, 103/146) was the predominant type, and isolates with PFGE patterns A and C were found in all four sampling areas. The isolates with patterns B and D were distributed in three of the four areas of Guangdong. The unique isolates with patterns F and G were each isolated from a single area. The archived studies showed that patterns D and F were the predominant genotypes in acute infections of juvenile tilapia in Guangdong and Guangxi and that the patterns A, B and E were mainly found in Fujian^16^. Thus, different from what had been previously found in juvenile tilapia, PFGE pattern C was the major prevalent pattern in inapparent infected adult/commercial tilapia in Guangdong, China. However, we wondered whether the PFGE patterns were linked to different pathogenesis in the strains isolated from juvenile and adult/commercial tilapia, particularly between patterns C and D/F. Pereira *et al.*^6^, using a tilapia infection model, showed that strains isolated from tilapia (with PFGE patterns A1, B, C, D and E) were highly virulent, causing 100% mortality in the tilapia. Moreover, they showed that the pathogenesis caused by the *S. agalactiae* human strains was not associated with a particular PFGE type. Therefore, they speculated that the fish strains from all genetic clusters were highly virulent in tilapia. However, we found that all of the strains identified in that study were obtained during outbreaks of meningoencephalitis and septicaemia at nine Nile tilapia farms. We believe that the pathogenesis caused by these outbreak strains (which were mostly isolated from acute infections in juvenile tilapia) was probably different from the pathogenesis of strains isolated from inapparent infected adult/commercial tilapias. Previous studies have shown that outbreaks of *S. agalactiae* infection in juvenile tilapia manifest with typical clinical signs, including exophthalmia, corneal opacity, swimming abnormalities and melanosis, and are associated with reduced survival conditions of tilapia, such as high temperature, ammonia, nitrate and low pH levels, in tilapia fish farms^34^. In contrast, no clinical symptoms were observed in the inapparent infected adult/commercial tilapia that were infected with *S. agalactiae* in this study. We therefore hypothesized that the *S. agalactiae* isolates that were identified in the present study had adapted to and were highly resistant to osmotic pressure, acidity and other environmental factors that are characteristic of the water body in fish farms. These would definitely induce resistance to immune responses in tilapia and result in unapparent infections in adult/commercial tilapia. Indeed, in resistance tests for osmotic pressure and temperature, the pattern C infective strains showed a higher resistance than did the pattern D strains, although they shared the same serotype and MLST type. However, Li *et al.*^18^ assumed that *S. agalactiae* develop highly efficient mechanisms to escape recognition and elimination by the host immune system, resulting in chronic streptococcosis. Therefore, the mechanism underlying these infections requires further investigation. Nevertheless, the archived studies indicate that epidemiological changes have likely contributed to the adaptation or transmission of particular *S. agalactiae* genotypes^38^ and that continuous surveillance for the prevalent *S. agalactiae* strains in adult/commercial tilapia would definitely provide a scientific basis for preventing and controlling tilapia streptococci diseases, which are a threat to occupational workers and customers around the world.

In conclusion, we have demonstrated that the prevalent strains that contribute to inapparent infected adult/commercial tilapia in Guangdong, China are *S. agalactiae*. Serotyping and MLST data suggest that there has been a clonal expansion of *S. agalactiae*. However, the joint assay to determine virulence gene profiles, PFGE patterns and geographical distribution data indicate that the unique serotype Ia (ST 7) is associated with a different genetic background in each area. The isolates with the PFGE pattern C, but not those with pattern D (which is frequently isolated during outbreak events), were the major prevalent *S. agalactiae* strains in inapparent infected adult/commercial tilapia in Guangdong, China.

## Additional Information

**How to cite this article**: Sun, J. *et al.* Inapparent *Streptococcus agalactiae* infection in adult/commercial tilapia. *Sci. Rep.* **6**, 26319; doi: 10.1038/srep26319 (2016).

## Supplementary Material

Supplementary Table S1

Supplementary Table S2

## Figures and Tables

**Table 1:** The primers used for specific PCR amplification in this study.

Gene	Primers	Nucleotide sequence (5′-3′)
16S rDNA	P1	CTAGAGTAC ACATGTACTTAAG
16S rDNA	P2	GGATTTTCCACTCCCATTAC
*cfb*	H1	AAGCGTGTATTCCAGATTTCCT
*cfb*	H2	CAGTAATCAAGCCCAGCAA

**Table 2:** The primers used for serotyping PCR amplification in this study.

Gene	Primers	Nucleotide sequence (5′-3′)
*cpsI*	cpsI-Ia-6-7-F	GAATTGATAACTTTTGTGGATTGCGATGA
*cpsI*	cpsI-6-R	CAATTCTGTCGGACTATCCTGATG
*cpsI*	cpsI-7-R	TGTCGCTTCCACACTGAGTGTTGA
*cpsI*	cpsL-F	CAATCCTAAGTATTTTCGGTTCATT
*cpsI*	cpsL-R	TAGGAACATGTTCATTAACATAGC
*cpsG*	cpsG-F	ACATGAACAGCAGTTCAACCGT
*cpsG*	cpsG-R	ATGCTCTCCAAACTGTTCTTGT
*cpsG*	cpsG-2-3-6-R	TCCATCTACATCTTCAATCCAAGC
*cpsN*	cpsN-5-F	ATGCAACCAAGTGATTATCATGTA
*cpsN*	cpsN-5-R	CTCTTCACTCTTTAGTGTAGGTAT
*cpsJ*	cpsJ-8-F	TATTTGGGAGGTAATCAAGAGACA
*cpsJ*	cpsJ-8-R	GTTTGGAGCATTCAAGATAACTCT
*cpsJ*	cpsJ-2-4-F	CATTTATTGATTCAGACGATTACATTGA

**Table 3:** The primers used for virulence gene determination in this study.

Gene	Primers	Nucleotide sequence (5′-3′)
*spb1*	*spb1*-F	GCTGAGACAGGGACAATTAC
	*spb1*-R	GTTGAAGGCAACTCAGTACC
*scpB*	*scpB*-F	ACAACGGAAGGCGCTACTGTTC
	*scpB*-R	ACCTGGTGTTTGACCTGAACTA
*bca*	*bca*-F	TAACAGTTATGATACTTCACAGAC
	*bca*-R	ACGACTTTCTTCCGTCCACTTAGG
*bac*	*bac*-F	CTATTTTTGATATTGACAATGCAA
	*bac*-R	GTCGTTACTTCCTTGAGATGTAAC
*dltR*	*dltR*-F	TTGACAGGTCTCTATGATTTAGTC
	*dltR*-R	GTCTGGTTCTCAGCCTAATTC
*cfb*	*cfb*-F	ATCGTTATGGTTTTTACATGA
	*cfb*-R	TTATTTTAATGCTGTTTGAAGTG
*sodA*	*soda*-F	GTAAAACGACGGCCAGT
	*soda*-R	AACAGCTATGACCATG

**Table 4:** The sampling areas and associated isolates in this study.

	Sampling numbers	Isolate numbers	Positive rate (%)
Maoming	68	52	76.47
Zhaoqing	14	0	0
Wuchuan	32	21	65.63
Zhuhai	85	49	57.65
Yangjiang	30	24	80.00
Total	229	146	63.76

**Table 5:** The correspondence between virulence gene profiles and PFGE patterns in this study.

Virulence gene profiles (strain number, percentage)	PFGE pattern
*dltr*-*bca*-*sodA*-*spb1*-*cfb*-*bac* (62, 42.47%),	A (7), B (10), C (44), D (1)
*dltr*-*bca*-*sodA*-*spb1*-*cfb*-*bac*-*scpb* (36, 26.47%)	A (5), B (4), C (26), D (1)
*dltr*-*bca*-*sodA*-*spb1*-*bac* (30, 20.55%)	A (3), B (2), C (21), D (3), F (1)
*dltr*-*bca*-*sodA*-*spb1*-*bac*-*scpb* (9, 6.16%)	A (1), C (6), D (1), G (1)
*dltr*-*bca*-*sodA*-*spb1* (4, 2.74%)	A (1), C (3)
*dltr*-*bca*- *spb1-bac* (1, 0.68%)	C (1)
*dltr*-*bca*-*sodA*-*bac* (1, 0.68%)	A (1)
*dltr*-*bca*-*sodA*-*bac-scpb* (1, 0.68%)	A (1)
*dltr*-*bca*-*sodA*-*cfb*-*bac*-*scpb* (1, 0.68%)	D (1)
*dltr*-*bca*-*sodA*-*spb1-scpb* (1, 0.68% )	C (1)

**Table 6:** The tests of resistance to osmotic pressure, acetic acid, and temperature in selected strains with each PFGE pattern.

Strain No.	PFGE pattern	NaCL (g/L)						Acetic acid (v/v,%)						Culture temperature (12 h)		Treatment (1 min)		
		5	10	20	30	40	50	1	3	5	10	20	30	45	65	80	90	95
GDSA001	A	+	+	+	+	—	—	±	—	—	—	—	—	+	—	—	—	—
GDSA003	A	+	+	+	—	—	—	+	—	—	—	—	—	+	—	—	—	—
GDSA065	A	+	+	+	+	+	—	±	—	—	—	—	—	+	+	—	—	—
GDSA093	A	+	+	+	+	—	—	±	—	—	—	—	—	+	—	—	—	—
GDSA158	A	+	+	+	—	—	—	—	—	—	—	—	—	+	+	—	—	—
GDSA050	B	+	+	+	—	—	—	+	—	—	—	—	—	+	—	—	—	—
GDSA055	B	+	+	+	+	—	—	±	—	—	—	—	—	+	—	—	—	—
GDSA058	B	+	+	+	+	—	—	+	—	—	—	—	—	+	—	—	—	—
GDSA071	B	+	+	+	+	—	—	+	—	—	—	—	—	+	—	—	—	—
GDSA004	C	+	+	+	+	+	—	—	—	—	—	—	—	+	+	—	—	—
GDSA090	C	+	+	+	+	±	—	±	—	—	—	—	—	+	±	—	—	—
GDSA095	C	+	+	+	+	+	—	—	—	—	—	—	—	+	+	—	—	—
GDSA113	C	+	+	+	+	+	—	—	—	—	—	—	—	+	+	—	—	—
GDSA147	C	+	+	+	+	±	—	+	—	—	—	—	—	+	±	—	—	—
GDSA051	D	+	+	+	+	±	—	+	—	—	—	—	—	+	±	—	—	—
GDSA056	D	+	+	+	+	—	—	+	—	—	—	—	—	+	—	—	—	—
GDSA131	D	+	+	+	+	—	—	—	—	—	—	—	—	+	—	—	—	—
GDSA136	D	+	+	+	+	—	—	—	—	—	—	—	—	+	—	—	—	—
